# Complimentary Role of [18F]FDG and [18F]NaF-PET/CT in Evaluating Synchronous Thyroid Carcinoma and Parathyroid Adenoma with Brown Tumors

**DOI:** 10.1055/s-0044-1787732

**Published:** 2024-06-14

**Authors:** Yeshwanth Edamadaka, Rahul V. Parghane, Sandip Basu

**Affiliations:** 1 Radiation Medicine Centre, Bhabha Atomic Research Centre, Tata Memorial Hospital, Mumbai, Maharashtra, India; 2 Homi Bhabha National Institute, Mumbai, Maharashtra, India

**Keywords:** brown tumor, [18F]FDG-PET/CT, [18F]NaF-PET/CT, parathyroid adenoma, papillary thyroid carcinoma, hyperparathyroidism, hungry bone syndrome

## Abstract

We herein present a patient initially suspected of multiple lytic skeletal metastasis of unknown primary on anatomical imaging. Metabolic imaging by [18F]-fluorodeoxyglucose (FDG) positron emission tomography/computed tomography (PET/CT) detected focal [18F]FDG uptake in the right thyroid nodule, mild [18F]FDG uptake in soft tissue lesion in the left inferior parathyroid region, and multiple nonavid osteolytic skeletal lesions. Fine-needle aspiration cytology of the right thyroid nodule showed papillary thyroid carcinoma (PTC). The patient had raised serum parathyroid hormone and serum calcium levels, suggesting parathyroid disease. [18F]-sodium fluoride (NaF)-PET/CT showed a metabolic superscan pattern of hyperparathyroidism with brown tumors rather than metastatic lytic skeletal lesions. Patient underwent total thyroidectomy and bilateral central compartment clearance, along with soft tissue lesion resection in the left inferior parathyroid region. Finally, histopathology confirmed PTC classical variant with no aggressive histology features (pT1N0) for thyroid nodule and parathyroid adenoma for soft tissue lesion in the left inferior parathyroid region. The findings of the [18F]FDG and [18F]NaF-PET/CT imaging were helpful for making a final diagnosis of synchronous thyroid cancer and parathyroid adenoma, which in turn guided the appropriate treatment strategy.

## Introduction

Brown tumor (BT) is a benign, reactive skeletal manifestation of long-standing hyperparathyroidism. 1 BT can affect any bone and radiologic findings are nonspecific, leading to misinterpretation. 2 Although the guidelines do not consider hyperparathyroidism a risk factor for thyroid carcinoma, it is not uncommon among hyperparathyroidism patients. Despite being benign, [18F]-fluorodeoxyglucose (FDG) positron emission tomography/computed tomography (PET/CT) shows increased [18F]FDG uptake in BT leading to uncertainty in concomitant thyroid carcinoma. 1 Carcinoma of unknown primary (CUP) accounts for approximately 0.5 to 9% of all malignancy and [18F]FDG-PET/CT demonstrates diagnostic importance over conventional imaging in determining primary tumor in CUP. 3 For multiple lytic skeletal lesions, the differential diagnoses include occult primary tumor, multiple myeloma, primary bone tumors, bone tuberculosis, and osteomyelitis. [18F]-sodium fluoride (NaF)-PET/CT can further help characterize skeletal lesions based on uptake pattern and its anatomical location and features. In this report, we present a case of synchronous thyroid carcinoma and parathyroid adenoma, wherein the utilization of [18F]FDG-PET/CT and [18F]NaF-PET/CT imaging helped in establishing the final diagnosis.

## Case Report

**Fig. 1:**
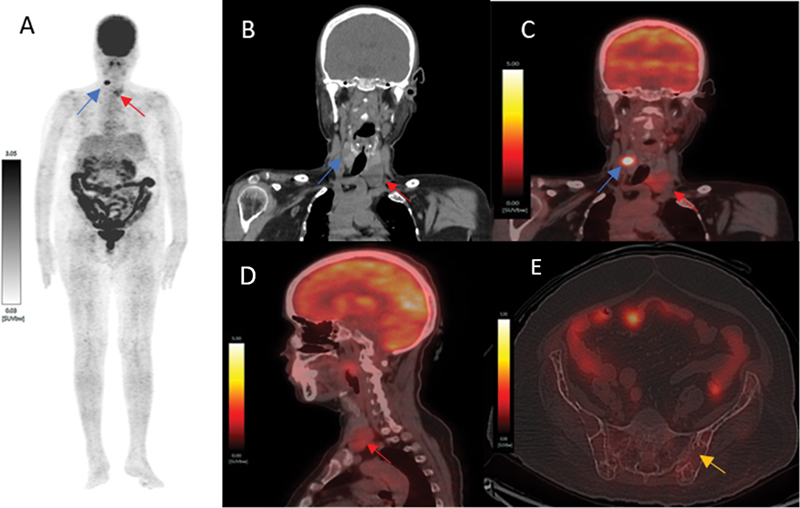
[18F]-fluorodeoxyglucose (FDG)-positron emission tomography/computed tomography (PET/CT) scan, ( **A** ) maximum intensity projection image (MIP), ( **B** ) coronal CT, and ( **C** ) fused coronal showed focal [18F]FDG avidity in the right thyroid nodule (blue arrow) and mild [18F]FDG uptake in soft tissue lesion in the left inferior parathyroid region (red arrow). ( **D** ) Sagittal fused image showed mildly [18F]FDG avid soft tissue lesion at posteroinferior to left thyroid lobe. ( **E** ) Axial fused image showed nonavid multiple osteolytic lesions in pelvis (yellow arrow).

**Fig. 2:**
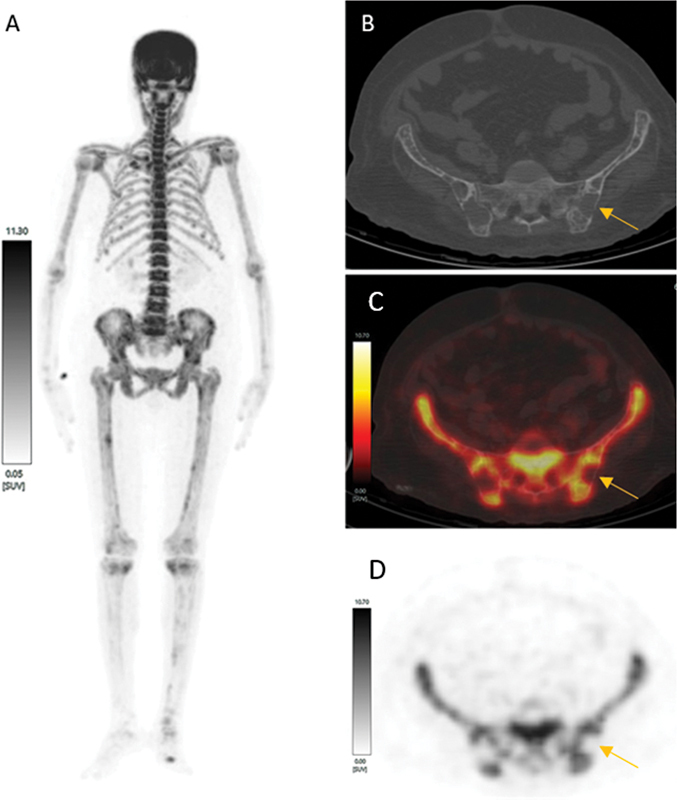
[18F]-sodium fluoride (NaF)-positron emission tomography/computed tomography (PET/CT), ( **A** ) maximum intensity projection image (MIP) image showed diffusely increased [18F]NaF uptake in axial and appendicular skeleton with no visualization of both kidneys suggestive of a metabolic superscan pattern. ( **B** ) Axial CT, ( **C** ) axial fused, and ( **D** ) axial PET images showed multiple osteolytic lesions in pelvis suggestive of brown tumors.

A 61-year-old female with abdomen-pelvic region pain and generalized bony pain for 2 years underwent contrast-enhanced CT scan revealing multiple osteolytic lesions in the pelvis raising suspicion of skeletal metastasis of unknown primary, among others. Patient underwent [18F]FDG-PET/CT which detected focal intense [18F]FDG uptake (maximum standardized uptake value [SUVmax] 9.0) in 0.9 × 0.8 cm sized right thyroid nodule and mild [18F]FDG uptake (SUVmax 2.5) in 2.8 × 2.2 cm sized soft tissue lesion in the posteroinferior region to left thyroid lobe and no abnormally increased [18F]FDG uptake in multiple osteolytic skeletal lesions ( Fig. 1 ). Fine-needle aspiration cytology (FNAC) from the right thyroid nodule lesion showed papillary thyroid carcinoma (PTC). Her biochemical analysis revealed elevated serum parathyroid hormone (PTH) of 742 pg/mL (10–55 pg/mL), serum calcium of 10.5 mg/dL (9.5–10.2 mg/dL), serum creatinine of 1.2 mg/dL (0.7–1.35 mg/dL), and serum alkaline phosphatase (ALP) of 1297 IU/L (44–147 IU/L), with no monoclonal band on serum electrophoresis ruling out multiple myeloma. She underwent total thyroidectomy and bilateral central compartment clearance, along with soft tissue lesion resection in the left parathyroid region. Intraoperatively, her serum PTH showed a marked reduction of 6.06 pg/mL. Histopathology confirmed PTC classical variant with no aggressive histological features like lymphovascular invasion or extrathyroidal extension and no evidence of lymph nodal involvement. Surgically resected thyroid lesion measured 1.2 × 1.0 × 1.0 cm (pT1N0). Resected soft tissue lesion in the left inferior parathyroid region showed circumscribed nodular lesion composed predominantly of chief cells in follicular pattern with reduced stromal adipocytes suggestive of left parathyroid adenoma. Postoperatively, patient had perioral numbness with low serum calcium of 6.4 mg/dL. Patient was treated with intravenous calcium gluconate thrice daily for 3 days and showed persistent low serum calcium levels of 6.9 mg/dL despite aggressive management suggestive of hungry bone syndrome (HBS). Her serum ALP level was still high (360 IU/L) and she was evaluated with [18F]NaF-PET/CT ( Fig. 2 ) to further characterize, which showed a metabolic superscan pattern with BT. Biopsy of the pelvic BT was not performed as it is invasive and considering old age with clinical context suggesting sequelae of hyperparathyroidism. Further management in this case includes replacement and supplementation of thyroxine and calcium. Routine biochemical measurements of serum calcium, PTH, and serum thyroglobulin as well as follow-up neck ultrasound examinations will be performed to monitor for recurrence of hyperparathyroidism and thyroid carcinoma, respectively.

## Discussion

In this report, [18F]FDG-PET/CT was initially ordered to find out the unknown primary in the patient with lytic skeletal lesions, which showed intense focal [18F]FDG uptake in the right thyroid nodule, mild [18F]FDG uptake in left inferior parathyroid adenoma, and no abnormally increased [18F]FDG uptake in multiple lytic skeletal lesions, which was unusual for skeletal metastasis needing further elucidation. Her biochemical analysis revealed hyperparathyroidism. [18F]NaF-PET/CT was performed which was suggestive of metabolic superscan pattern. 4 A focal [18F]FDG uptake in incidental thyroid lesions carry a high risk of malignancy, on the contrary the risk is low in the case of diffuse [18F]FDG uptake. 5 Incidental [18F]FDG avidity in parathyroid adenoma has been reported in literature. 6 Most of the reported cases include papillary microcarcinoma with similar incidence of primary (9.1%) and secondary (7.2%) hyperparathyroidism. 7 Similar to our case report, Thanseer et al demonstrated dual pathologies of PTC and parathyroid adenoma using dual PET tracers, illustrating intense [18F]FDG uptake in subcentimetric right thyroid nodule and [18F]-choline uptake in left parathyroid adenoma. 8

The standard nuclear medicine modalities for detecting parathyroid adenoma include [99m]Tc-sestamibi scintigraphy, which serves the need of a reliable localization of adenoma as prerequisite for minimal invasive surgery. The management of parathyroid adenoma with minimally invasive parathyroidectomy offers many advantages, but it is difficult to detect concomitant thyroid malignancy. Therefore, a thorough assessment of thyroid nodules using neck ultrasound and FNAC is useful to potentially diagnose synchronous presentations. 9 Long-standing parathyroid adenoma can be complicated by benign multiple lytic skeletal lesions that may morphologically resemble lytic skeletal metastases on anatomical imaging. Histopathological findings of BT include osteoclastic resorption with cavities filled with fibrous tissue, giant cells, hemosiderin deposits, and macrophages. 10

The role of [18F]FDG-PET/CT in detecting BT is limited to individual case reports showing variable [18F]FDG uptake in BT. 11 In our case report, BT showed no abnormal [18F]FDG uptake, a finding that has not been widely reported previously to our knowledge. To further investigate the pathology, a [18F]NaF-PET/CT was performed, which showed diffusely increased homogenous [18F]NaF uptake in the axial and appendicular skeleton including calvarium and distal extremities, with less background activity suggestive of a typical metabolic superscan pattern, while a metastatic superscan pattern would show heterogeneous axial and proximal appendicular skeleton. To summarize, [18F]NaF-PET/CT demonstrates bone remodeling caused by hyperparathyroidism of parathyroid adenoma leading to diffusely increased uptake in BT. 12

The postoperative course in hyperparathyroidism with multiple BTs can cause a sudden decline in serum calcium to a dangerous state called as HBS. HBS entails high bone turnover, necessitating intensive intravenous calcium replacement to prevent complications. HBS is defined as clinically symptomatic hypocalcemia without hypoparathyroidism. 13 This can cause prolonged hospitalization in patients after surgery requiring aggressive management. A study showed a direct correlation of total metabolic active bone volume using [18F]NaF-PET/CT with a duration of postoperative intravenous calcium substitution as PTH stimulates bone metabolism not only in BT but in the whole skeleton. 12 In our case report, a metabolic superscan pattern on [18F]NaF-PET/CT with low serum calcium level was found in the postoperative period suggestive of presence of HBS.

## Conclusion

The diagnosis and management of multiple osteolytic lesions present significant challenges due to the possibility of various clinical conditions, both malignant and benign, that can induce osteolysis in the skeleton. The presented case illustrates the useful role of dual tracer PET/CT imaging with [18F]FDG and [18F]NaF, in determining the cause of these conditions and subsequently directing the appropriate therapeutic approach.
